# A randomized cross over trial of tolerability and compliance of a micronutrient supplement with low iron separated from calcium vs high iron combined with calcium in pregnant women [ISRCTN56071145]

**DOI:** 10.1186/1471-2393-6-10

**Published:** 2006-04-04

**Authors:** Eric Ahn, Nicholas Pairaudeau, Nicholas Pairaudeau, Yves Cérat, Bernard Couturier, Andre Fortier, Éric Paradis, Gideon Koren

**Affiliations:** 1 The University of Toronto, Clinique de Gynécologie-Obstétrique Pierre Boucher, Longueuil, Québec, Canada; 2 Division of Clinical Pharmacology/Toxicology, The Hospital for Sick Children, North York General Hospital, Toronto, Canada

## Abstract

**Background:**

Prenatal micronutrient combinations with high iron content are associated with high rates of gastrointestinal symptoms. This coupled with nausea and vomiting of pregnancy results in women often discontinuing their multivitamins.

A new prescription supplement (PregVit^®^) that separates iron from calcium in two tablets – morning and evening, has lower elemental iron content (35 mg), but results in similar extent of iron absorption when compared to another supplement containing (60 mg) of elemental iron (Materna^®^). The objectives of this study were to compare tolerability and compliance with PregVit^® ^vs. a supplement with high iron content (Materna^®^), in pregnant women.

**Methods:**

Randomized, crossover open labeled study in 135 pregnant women attending outpatient clinics in Ontario and Quebec.

**Results:**

Use of PregVit^® ^was associated with a 30% reduction in constipation rate as compared to Materna^®^. Both products demonstrated similar compliance rates.

Compliance of Materna^® ^was negatively associated with the severity of nausea and vomiting of pregnancy. No such correlation was found for PregVvit^®^.

**Conclusion:**

PregVit^®^, a supplement with lower iron content (35 mg), has significantly decreased constipation rates as compared to 60 mg iron- Materna and has similar compliance rates. High iron content in multivitamin supplements is associated with adverse effects in pregnancy.

## Background

Prenatal micronutrient supplementation is indicated for all pregnant women, because the recommended daily allowances (RDA) for many vitamins and minerals are not met during pregnancy with dietary sources alone [1,2]. Prior to 1990's, physicians typically commenced prenatal micronutrients at the time of diagnosis of pregnancy. However, proving the ability of folic acid supplementation to prevent neural tube defects, has led to the recommendation to start prenatal micronutrient supplementation when planning pregnancy [3,4]. A recent analysis has further documented the importance of micronutrient supplementation during pregnancy in preventing malformations other than neural tube defects [5]. The findings are consistent with the notion that periconceptional multivitamin use, containing folic acid, reduces the overall occurrence of birth defects by 15–33%, in addition to the demonstrated reduction in neural tube defects [6].

Typically, many prenatal supplements contain, among other minerals, elemental iron at 50–60 mg, to prevent pregnancy-induced anemia secondary to increasing fetal needs. However, this level of iron content has been associated adverse gastrointestinal effects, including nausea, vomiting and constipation. The common occurrence of nausea and vomiting of pregnancy (NVP) has further led women to discontinue prenatal supplementation containing these levels of iron [7].

A new prescription prenatal micronutrient supplement PregVit^® ^has recently been introduced into the Canadian Market aiming to address these issues: PregVit^®^, taken twice daily, contains less elemental iron (35 mg) than many other prenatal supplements. Iron is incorporated in the morning dose (Table 1). The evening dose of PregVit^® ^contains calcium, which is known to inhibit iron absorption from the gastrointestinal tract [8-11]. By separating the two elements it was hypothesized that despite a lower total iron dose – a similar systemic exposure to iron would occur as with the other high iron prenatal supplements. Moreover, PregVit^® ^contains higher doses of vitamin C, which is also known to facilitate iron absorption (Table 1).

**Table 1:** Composition of two prenatal supplements and Recommended Dietary Allowance (RDA) values^12^

**Component**	**PregVit**^®^	**Materna**^® ^***	**Recommended dietary allowance (RDA)**
	a.m. tablet**		
Vit. A^ψ^	**2700 IU (β-Carotene)**	**1500 IU (β-Carotene) 1500 IU (acetate)**	**750–770 μg retinol**
Vit. E^ξ^	**30 IU**	**30 IU**	**15 mg**
Vit. C	**120 mg**	**100 mg**	**80–85 mg**
Vit. B_1 _(thiamin)	**3 mg**	**3 mg**	**1.4 mg**
Vit. B_2 _(riboflavin)	**3.4 mg**	**3.4 mg**	**1.4 mg**
Niacinamide	**20 mg**	**20 mg**	**18 mg**
Vit. B_6_	**10 mg**	**10 mg**	**1.9 mg**
Pantothenic acid (calcium pantothenate)	**5 mg**	**10 mg**	6 mg*
Magnesium	**50 mg**	**50 mg**	**350–400 mg**
Iodine	**0.15 mg**	**0.15 mg**	**0.22 mg**
Iron	**35 mg (as fumarate)**	**60 mg (as fumarate)**	**27 mg**
Copper	**2 mg**	**2 mg**	**1 mg**
Zinc	**15 mg**	**25 mg**	**11–12 mg**
	p.m. tablet**		
Folic Acid	**1.1 mg**	**1 mg**	**0.6 mg**
Vit. B_12 _(cyanocaobalamin)	**12 μg**	**12 μg**	**2.6 μg**
Vit. D (cholecalciferol)^ε^	**250 IU**	**250 IU**	5 μg*
Calcium	**300 mg**	**250 mg**	1000–1300 mg*
Biotin	**0**	**30 μg**	**-**
Chromium	**0**	**25 μg**	29–30 μg*
Manganese	**0**	**5 mg**	2 mg*
Molybdenum	**0**	**25 μg**	**50 μg**
Selenium	**0**	**25 μg**	**60 μg**
Pill Size	**Small**	**large**	

* **Adequate Intake**

** **Applies only to PregVit**^®^

^ψ^**VIT A **– 1 International Unit (IU) of retinol is equivalent to 0.3 μg retinol (750 μg of retinol is 2,500 IU of retinol)

2 μg of supplemental β-carotene is equivalent to 1 μg retinol ^3 (page 92 & 565)^

^ξ^**VIT E **– 1 International Unite (IU) is equivalent to 0.67 μg of α-tocopherol (15 mg is equivalent to 22,388 IU)

However, different conversion factors are used for different forms of vitamin E

ε**VIT D **– 1 IU is equivalent to 0.025 μg of cholecalciferol (5 μg is equivalent to 200 IU)

***Please note: Since the completion of this study a new formulation of Materna was introduced containing 27 mg of elemental iron.

A recent pharmacokinetic study has shown that the extent of iron absorption with PregVit^® ^(in terms of area under the concentration-time curve) was similar to that of Materna^®^, despite PregVit^®^containing only about half of the iron dose [12].

The objective of the present study was to compare tolerability and compliance with PregVit^® ^and Materna^® ^among pregnant women.

## Methods

Approval of the study protocol was obtained from the local ethics committee at The Hospital for Sick Children, in Toronto, Ontario, the North York General Hospital (NYGH) in Toronto, Ontario and the Clinique de Gynécologie-Obstétrique Pierre Boucher (CGOPB) in Quebec. Appropriate sample size was determined to be one hundred participants based on a desired effect size of 20% fewer adverse effects with PregVit^® ^versus Materna^®^.

Participants were eligible to enroll in the study if they were pregnant and between 18 and 45 years of age. This was a prospective, randomized, open labeled, cross over study. Between June 2003 and November 2003, women attending out patients obstetric clinics at NYGH and CGOPB were invited to enroll in the study. During their regular first obstetric appointment, participants reviewed the protocol and signed an informed consent form. Participating women were asked to complete a standardized questionnaire recording demographic data, pregnancy and, medical history, drug and vitamin use and information on their normal bowel movements. Based on the existence of NVP, participants were block-randomized to receive either Materna^® ^or PregVit^® ^to be taken for one month. The block randomization aimed at ensuring similar numbers of women with NVP/no NVP receiving each of the products first as NVP may affect compliance. They were asked to keep a diary of any adverse event (*e.g*. constipation, nausea and headache). The severity of their NVP was monitored by the Pregnancy-Unique Questionnaire of Emesis and nausea (PUQE) scores (Table 2) [13]. In addition, changes in diet as well as the use of medications and adherence to the study drug were recorded in the diary. At the end of the month a pill count was conducted to corroborate their diary reports. One month later, upon their regular second follow-up visit, patients returned their diaries and pill bottles. The women were then given the alternative product and were asked to record the same information in the diary for the following month. Upon completion of the two month cross over, participants were asked to return their diaries and supplement bottles for a pill count.

**Table 2:** The PUQE Score (12). Patients were asked to circle the appropriate box next to the three symptoms of nausea and vomiting of pregnancy (NVP). The score of each box is represented by the top row. The total score is calculated and determines the severity of NVP^14^. A score of 3 to 6 represents Mild NVP. A score of 7 to 12 indicates Moderate NVP. Finally a score of 13 and greater represents Severe NVP.

	**1**	**2**	**3**	**4**	**5**
***QUESTION 1***In the last 12 hours, how long have you felt **nauseated or sick at your stomach**?	Not at all	1 hour or less	2–3 hours	4–6 hours	More than 6 hours (Please specify number of hours)
***QUESTION 2***In the last 12 hours, have you **vomited or thrown up**?	I did not throw up	1–2 times	3–4 times	5–6 times	7 or more times (Please specify number of times)
***QUESTION 3***In the last 12 hours, **how many **times have you had **retching or dry heaves **without bringing anything up?	No Time	1–2 times	3–4 times	5–6 times	7 or more (Please specify number of times)

Before starting the study, a pilot sample of 10 adult volunteers was tested for the feasibility of completing the diary. They took PregVit^® ^for one week followed by Materna^® ^for one week and were asked to fill out the diary. Minor changes were made to the diary based on comments collected during the pilot phase.

### Sample size and statistical analysis

The primary end point of interest was the mean rate of adverse events in the two study arms, including decreased compliance. The mean compliance based on pill counts and diary reports were compared between the two study arms by paired Student's *t *test or signed rank test, as appropriate. Other variables such as mean days of experiencing adverse drug event (ADE), nausea, constipation and other ADE, excluding nausea and constipation, (labeled as *other*), were also compared using the same statistical tests, as appropriate. The incidence of nausea and noncompliance due to constipation were compared between the two groups using chi-square. All statistical tests were performed using Sigmastat^® ^statistical software, Version 2.03 (SPSS Inc, Chicago, IL). Subsequently, multilinear regression analysis was conducted to elucidate determinants that affected compliance rate with the study drugs. Determinants of compliance were chosen based on factors showing significant in recent literature [14-17].

Rates of constipation were the primary end point of interest, as high iron content is known to induce constipation. Sample size of 100 was calculated to show a 33% decrease in rate of constipation (from 30% to 20%) at power of 80% and alpha of 5%.

## Results

A total of 109 patients from NYGH consented to enroll in the study. Of these 109 patients, 37 patients with NVP and 45 Non NVP patients completed both arms of the study, totaling 82 patients (75.2%). Of 100 patients agreed to enroll in the study at the CGOPB (35 NVP and 65 Non NVP), 18 NVP patients and 38 Non NVP patients, for a total of 56 patients, completed the study (56%). Hence, a total of 138 patients completed the study and were included in the data analysis. The mean gestational age at the start was 8 ± 2 weeks. There was identical gestational age at start in the two arms. The women who dropped out from the study almost entirely due to the fact that they did not return for follow-up or did not fill out diaries on returning the pill bottles. They did not differ from those who completed the study in any characteristic collected and reported by us. Among those who did not complete the study, there was no higher prevalence to any of the two arms (*e.g*., PregVit^® ^vs Materna^®^).

There was a significantly higher incidence of reported constipation, as well as average duration of constipation when taking Materna^® ^as compared to PregVit^® ^(Table 3).

**Table 3:** Overall study results, comparing Pregvit^® ^(low iron) to Materna^® ^(high iron) over a month administration in a cross-over design.

	(n)*	**PregVit^® ^(SD)**	**Materna^® ^(SD)**	**p value**
**Pill Count (% of 100%)**	78	87.7 ± 20	90.9 ± 17	0.11
**Reported Compliance (%)**	138	90.4 ± 18	92.4 ± 15	0.42
**Adverse events (%)**	138	17.6 ± .24	20.3 ± 24	0.14
**Nausea (%)**	138	9.3 ± 19	10.1 ± 18	0.71
**Constipation time length (%)**	138	3.1 ± 8	4.7 ± 11	**0.05**
**Other adverse events**	138 **(n)**	10.4 ± 21	99.9 ± 20	0.95
**Nausea rate (%)**	138	41.3 (57/81)	45.7 (63/75)	0.54
**Constipation Rate (%)**	138	22.5% (31/107)	34.8% (48/90)	**0.03**

**n **represents the number of patients (out of 138) who completed any particular item.

Multivariate linear regression was conducted to elucidate factors that independently affected compliance. The factors tested included: age, education, province, marital status, weight, NVP severity, any substance used such as illicit drugs and multivitamin supplement (which multivitamin they started the trial with). These factors have been shown in previous studies to affect compliance [18]. The dependent variable used was reported compliance. Including all 138 patients, using both arms of the study, no variable significantly predicted compliance rate (Table 4).

**Table 4:** Multiple linear regression of factors affecting compliance in both treatment arms.

**Independent Variable**	**Coefficient**	**Standard Error**	**p value**
Age	0.00	0.002	0.43
Education	-0.01	0.011	0.58
Province	0.04	0.026	0.17
Marital Status	-0.02	0.028	0.43
Weight	0.00	0.000	0.14
NVP Severity	-0.02	0.012	0.08
Substance use*	0.00	0.036	0.79
Multivitamin Supplement	-0.01	0.019	0.75

* alcohol, tobacco or illicit drugs

Finally, multiple linear regression with patients and their reported compliance while taking Materna^® ^revealed that marital status and NVP severity predicted their reported compliance; single motherhood predicted higher compliance rates whereas more severe NVP predicted a lower compliance rate (Table 5). No such correlation was detected for PregVit^®^.

**Table 5:** Multiple linear regression of compliance in patients while taking Materna^®^

**Independent Variable**	**Coefficient**	**Standard Error**	**p value**
Age	0.00	0.002	0.73
Education	-0.02	0.014	0.09
Province	0.03	0.031	0.37
Race	0.02	0.010	0.11
Marital Status	-0.08	0.034	0.02
Weight	0.00	0.000	0.14
NVP Severity*	-0.05	0.014	0.002
Substance**	-0.06	0.044	0.20

* More severe NVP was associated with significantly lower compliance. No such effect was found with Pregvit^®^, which is a substantially smaller tablet, containing much lower iron content.

** alcohol, tobacco or illicit drugs

## Discussion

The results of this study demonstrate that pregnant women may experience less constipation when taking PregVit^®^. These results are likely attributed to the lower iron dose contained in PregVit^® ^relative to Materna^® ^(35 mg and 60 mg, respectively) (Table 1). The other differences in micronutrient content between the two preparations are not known to affect constipation rate. It should be noted that since the completion of this study the dose of iron in Materna^® ^has been reduced to 27 mg. The reformulated Materna^® ^has the same levels of calcium, manganese and zinc as before. Thus, with lower vitamin C content than PregVit^®^, it is possible that the systemic absorption of iron of Materna^® ^may be subtherapeutic^14^. Moreover, studies have suggested an antagonistic relationship between iron and zinc and manganese, in which zinc and manganese reduce the positive effects of iron supplementation and vice versa [19,21].

It is likely that the higher dose of zinc, found in Materna^® ^versus PregVit^® ^plus manganese, (which is not included in PregVit^®^) all interfere with the absorption of iron. A 1993 study(16) found that supplementation with a low daily dose (18 mg) of iron given in a prenatal supplement together with inhibitive minerals was not sufficient to cover the iron needs of most pregnant women. Importantly, many prenatal supplements currently available still contain 50–60 mg of elemental iron.

Two methods were used to estimate compliance: Patients' reported compliance and pill count. Using these measures, compliance was found to be similar for both products. The similar compliance may be a reflection of better tolerability with PregVit^®^, being partially offset by the need to take this supplement twice daily, as compared to once daily with Materna^®^.

Of potential clinical importance, the multivariate analysis revealed that compliance with Materna^® ^was negatively related to the severity of nausea and vomiting of pregnancy. This agrees with a recent study documenting that the severity of morning sickness adversely affects use of Materna^® ^(6). This may be explained also by a substantially larger pill size as compared to PregVit^®^, in addition to the direct effect of iron. These findings suggest that PregVit^® ^may confer an advantage to women suffering from NVP due to both higher tolerability because of lower iron content and a smaller tablet size. Because NVP can very from slight food aversion to hyperemesis gravidarum, we used a validated tool that quantifies the severity of NVP (Table 2).

Potential weaknesses of this study are that the participation in this cross over protocol may act to improve compliance among participant, and that, similar to other randomized controlled studies, the demonstrated high compliance figures do not reflect those of naturalistic "real life" drug administration. We experienced a relatively high drop out rate. This did not reflect failure to take the two products but rather lack of motivation of healthy pregnant women to participate in this type of intensive protocol including daily diaries and returning pills. However, because each woman served as her own control, the attrition does not bias the results. The women who dropped out were not different in their characteristics from those who completed the protocol. Importantly, because compliance rate was an important endpoint we refrained from contacting the women to remind them of their participation, an act that could artificially affect compliance, with the medications. Previous studies also reported on diarrhea occurring with micronutrients, however, in our study the occurrence of diarrhea was very rare and not different between the two study interventions.

Yet, this study, comparing tolerability and compliance rates between two prenatal micronutrient supplements different in their iron content and administration schedule, has identified several determinants of potential clinical significance, which may affect pregnant women's adherence to micronutrient supplementation.

A major problem in primary prevention of congenital anomalies by folic acid or folic acid containing micronutrients is the need to commence supplementation preconceptionally. There is a serious and urgent need to educate potential parents to start such supplementation before pregnancy is confirmed.

## Conclusion

PregVit^®^, a supplement with lower iron content (35 mg), has significantly decreased constipation rates as compared to 60 mg iron- Materna and has similar compliance rates. High iron content in multivitamin supplements is associated with adverse effects in pregnancy.

## Competing interests

G. Koren has been a paid consultant for Duchesnay Inc. All other authors declare they have no competing interests'.

## Authors' contributions

Eric Ahn collected all data in Toronto, analyzed the data and wrote the draft manuscript.

Nicholas Pairaudeau oversaw the study in the Toronto site.

Nicholas Pairaudeau Jr. participated in collection and analysis of data.

Yves Cerat, B. Couturier, A. Fortier and E. Paradis oversaw patients and collected the data in the Quebec Site.

Gideon Koren wrote the protocol and oversaw all stages of collection, analysis and writing.

## Pre-publication history

The pre-publication history for this paper can be accessed here:


